# The Late Dr. Conolly, of Hanwell, England

**Published:** 1871-09

**Authors:** 


*The late Dr. John Conolly, of Hanwell, England.* By CHARLES A. LEE, M.D.

This is an able sketch of the life of Dr. Conolly, who was one of the first advocates of non-restraint treatments in the managing of the insane. Though dead some years, yet his teachings deserve rememberance, and Prof. Lee has contributed wisely and liberally to this object.

